# Fucoidan Attenuates Cardiac Remodeling by Inhibiting Galectin-3 Secretion, Fibrosis, and Inflammation in a Mouse Model of Pressure Overload

**DOI:** 10.3390/biomedicines12122847

**Published:** 2024-12-14

**Authors:** Wen-Rui Hao, Chun-Han Cheng, Huan-Yuan Chen, Tzu-Hurng Cheng, Ju-Chi Liu, Jin-Jer Chen

**Affiliations:** 1 Division of Cardiology, Department of Internal Medicine, Shuang Ho Hospital, Ministry of Health and Welfare, Taipei Medical University, New Taipei City 23561, Taiwan; b8501043@tmu.edu.tw; 2 Division of Cardiology, Department of Internal Medicine, School of Medicine, College of Medicine, Taipei Medical University, Taipei City 11002, Taiwan; 3 Department of Medical Education, Linkou Chang Gung Memorial Hospital, Taoyuan City 33305, Taiwan; leocheng1991@gmail.com; 4 Institute of Biomedical Sciences, Academia Sinica, Taipei City 115201, Taiwan; hchen9@ibms.sinica.edu.tw (H.-Y.C.); jc8510@yahoo.com (J.-J.C.); 5 Department of Biochemistry, School of Medicine, College of Medicine, China Medical University, Taichung 404328, Taiwan; 6 Division of Cardiology, Department of Internal Medicine and Graduate Institute of Clinical Medical Science, China Medical University, Taichung City 404328, Taiwan

**Keywords:** fucoidan, galectin-3, pressure overload, fibrosis

## Abstract

Background/Objectives: Fucoidan, a sulfated polysaccharide derived from marine algae, is known for its antioxidant and immunomodulatory properties. Galectin-3 (Gal-3), a protein associated with cardiovascular fibrosis, has been identified as a potential therapeutic target in cardiac remodeling. This study aimed to evaluate whether fucoidan could inhibit Gal-3 activity and mitigate cardiac remodeling in a mouse model of pressure overload-induced cardiac hypertrophy. Methods: To test this hypothesis, we used transverse aortic constriction (TAC) surgery to induce pressure overload in normotensive mice, replicating the pathological features of cardiac hypertrophy. Mice were treated with fucoidan at a dose of 1.5 or 7.5 mg/kg/day. In vivo assessments of cardiac function, fibrosis, inflammation, and Gal-3 expression were performed. Results: Pressure overload led to significant upregulation of serum Gal-3 levels, increased cardiac collagen deposition, and elevated markers of fibrosis and inflammation. In mice treated with fucoidan, these effects were significantly attenuated. Fucoidan treatment prevented the upregulation of Gal-3, reduced collagen deposition, and decreased inflammatory cell infiltration, suggesting an inhibition of both fibrosis and inflammation. Conclusions: Fucoidan effectively mitigated the adverse effects of pressure overload in this mouse model, including reduced Gal-3 expression, fibrosis, and inflammation. These findings suggest that fucoidan holds promise as a therapeutic agent for preventing or delaying cardiac remodeling and associated complications, such as fibrosis and inflammation, in pressure overload-induced cardiac hypertrophy. Further research is needed to explore the underlying mechanisms and clinical applicability of fucoidan in cardiac disease.

## 1. Introduction

Cardiac remodeling is a pivotal process underlying the progression of cardiovascular diseases, often leading to heart failure (HF). This process involves structural and functional adaptations in response to stressors such as hypertension, which initially serve as compensatory mechanisms but ultimately lead to pathological changes, including increased myocardial stiffness and reduced cardiac efficiency [1,2]. Galectin-3 (Gal-3), a β-galactoside-binding lectin, has emerged as a key mediator in cardiac remodeling. It influences inflammation, fibrosis, and extracellular matrix remodeling through its interactions with cellular receptors and signaling pathways [3,4]. Elevated Gal-3 levels are associated with adverse cardiac outcomes, including fibrosis and inflammation, making it a potential therapeutic target [5,6]. Prior studies have demonstrated that blocking Gal-3 can attenuate myocardial damage in ischemia/reperfusion injury and improve cardiac outcomes [7]. Fucoidan, a sulfated polysaccharide derived from brown algae, has shown potential as a cardioprotective agent due to its anti-inflammatory, antioxidative, and antifibrotic properties. Its ability to modulate key pathways, such as the nuclear factor E2 related factor 2 (Nrf2)/glutathione peroxidase 4 (GPX4) axis and CD44 signaling, helps mitigate oxidative stress and inflammatory responses, which are critical in cardiac remodeling [8,9]. In preclinical models, fucoidan has been shown to reduce fibrosis, suppress inflammation, and improve cardiac function, suggesting its potential to complement Gal-3-targeted therapies [10,11]. Given these insights, targeting maladaptive remodeling through fucoidan offers a promising strategy for HF management. This study aims to investigate the therapeutic effects of fucoidan in a pressure-overload-induced cardiac remodeling model, with a focus on its interaction with Gal-3 and its potential to modulate inflammatory and fibrotic responses.

## 2. Materials and Methods

### 2.1. Materials

Fucoidan, a sulfated polysaccharide, was sourced from Hi-Q (OliFuco^®^ Plus). Primary antibodies used in this study were directed against several key proteins, including Gal-3 (R&D, Cat number MAB1197); CD68 (Bioss, Cat number bs-0649R); connective tissue growth factor (CTGF) (Santa Cruz, Cat number sc-14939); discoidin domain receptor 2 (DDR2) (Santa Cruz, Cat number sc-8989); alpha-smooth muscle actin (alpha-SMA) (abcam^®^, Cat number ab5694); and CD44 (MCE, Cat number HY-P80062). Additionally, collagen I (Santa Cruz, Cat number sc-59772) was detected using primary antibodies. GAPDH (Cell Signaling, Cat number 2118) was utilized as a loading control. Secondary antibodies included horseradish peroxidase (HRP)-conjugated anti-mouse IgG (BioLegend, Cat number B426166), a common choice for enhanced signal detection via chemiluminescence. Protein quantification was conducted using the Bicinchoninic Acid (BCA) Protein Assay Kit (Thermo, Cat number 23228), a standard approach for determining protein concentration. For chemiluminescent visualization of Western blot results, Immobilon Western Chemiluminescent HRP Substrate from Millipore Corp (Billerica, MA, USA) was employed, allowing sensitive detection of HRP-conjugated secondary antibodies. Histological analysis employed Sirius red staining (SIGMA, Cat number 365548) and Masson Trichrome Staining Kits (SIGMA, Cat number 41116121), which, respectively, enabled the identification of cellular and fibrotic changes within the tissue samples. 

### 2.2. Animals and Animal Models

**Figure 1:**
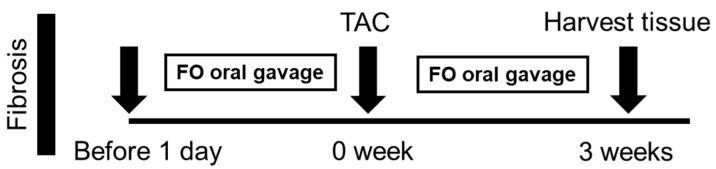
Timeline of fucoidan (FO) administration and tissue collection in TAC-induced cardiac remodeling model. Male C57BL/6J mice (8–10 weeks, 23.5–27.5 g) underwent TAC surgery to induce cardiac hypertrophy and fibrosis or sham surgery as a control. Mice received FO by oral gavage starting one day before TAC surgery and continuing daily until tissue collection at 3 weeks post-surgery. FO was administered in two doses: low-dose fucoidan (FOL; 60 mg/kg/day, equivalent to 1.5 mg/day for a 25 g mouse) and high-dose fucoidan (FOH; 300 mg/kg/day, equivalent to 7.5 mg/day for a 25 g mouse) for 14 days. The arrow represents the timeline of the study, indicating the duration and sequence of fucoidan administration and tissue collection. At the end of the experiment, tissues were harvested to assess the effects of FO on cardiac remodeling.

All animal experiments adhered to the Guidelines for the Care and Use of Laboratory Animals by the U.S. National Institutes of Health (NIH Publication, revised 2011) and received approval from the Laboratory Animal Welfare and Ethics Committee at Taipei Medical University. Male C57BL/6J mice, aged 8–10 weeks and weighing between 23.5 and 27.5 g, were obtained from the Institute of Laboratory Animal Science, Academia Sinica, Taipei, Taiwan. To investigate the effects of fucoidan on transverse aortic constriction (TAC), mice were subjected to either a sham procedure, TAC surgery, or TAC surgery followed by treatment with 1.5 mg/kg/day fucoidan (FOL) or 7.5 mg/kg/day fucoidan (FOH). These procedures followed established protocols that reliably model pressure overload-induced cardiac hypertrophy and heart failure in vivo. The overall animal treatment protocol is illustrated in Figure 1. For surgical preparation, anesthesia was induced via intraperitoneal injection of 80 mg/kg pentobarbital sodium (3% concentration; SIGMA, Cat number T48402), providing adequate depth for thoracic procedures. After induction, each mouse was intubated to secure the airway and mechanically ventilated with settings adjusted to 13–15 cmH_2_O pressure and 105 breaths per minute to maintain stable respiration. The surgical site was sterilized to minimize infection risk, and a 5 mm incision was made at the left second or third intercostal space. Underlying muscle and soft tissue were carefully dissected to expose the thoracic cavity. After entering the thoracic cavity, the intercostal muscle was separated approximately 2 mm from the sternum, and the chest cavity was expanded by 5 mm with a small chest retractor, allowing adequate visualization of the descending aorta. To achieve aortic constriction, a 6-0 surgical suture was looped around the thoracic aorta to simulate pressure overload, and the ligature was tied securely before the pad needle was withdrawn. The thoracic cavity and skin were then closed layer by layer to maintain anatomical integrity. Postoperative care included recovery monitoring in a warmed environment. In sham-operated control mice, the procedure was identical except that the aorta was not ligated, providing baseline measurements for comparison.

### 2.3. Echocardiography and Hemodynamics

In this study, transthoracic echocardiography was performed to evaluate cardiac structure and function in mice subjected to either sham or TAC surgery. Using a Philip IE-33 high-resolution imaging system equipped with a 25 MHz RMV-710 transducer, key parameters—including left ventricular end-diastolic diameter (LVEDd), left ventricular end-systolic diameter (LVESd), and left ventricular ejection fraction (LVEF)—were measured at the mid-papillary level from M-mode tracings. These metrics provided detailed insights into ventricular remodeling and systolic function under pressure overload conditions. To ensure reproducibility and reliability of echocardiographic measurements, inter- and intra-observer variability coefficients were assessed. Inter-observer variability, determined by two independent investigators analyzing the same set of echocardiograms, yielded a coefficient of variation of 5.2% for LVEDd, 6.1% for LVESd, and 4.8% for LVEF. Intra-observer variability, assessed by reanalyzing the same images after a two-week interval, showed coefficients of variation of 4.6% for LVEDd, 5.3% for LVESd, and 4.2% for LVEF. These low variability values underscore the consistency and accuracy of the echocardiographic data. In addition to echocardiography, hemodynamic parameters were assessed via cardiac catheterization. Mice were anesthetized using 1.5% isoflurane to maintain stable physiological conditions. A microtip catheter transducer was introduced through the right carotid artery into the left ventricle to continuously monitor pressure and heart rate. Hemodynamic data, including left ventricular systolic pressure (LVSP) and the maximum and minimum rates of pressure change (dP/dt_max and dP/dt_min), were analyzed using LabChart 7 software. This approach provided a high-resolution evaluation of cardiac performance, capturing both structural and functional responses to pressure overload. The combined use of echocardiography and invasive hemodynamic measurements offers a comprehensive assessment of cardiac remodeling and function. The integration of inter- and intra-observer variability coefficients ensures transparency and underscores the robustness of the echocardiographic methodology employed in this study.

### 2.4. Histological Analysis

Cardiac tissue samples were processed through a comprehensive series of steps to assess histopathological changes and collagen deposition accurately. Initially, the tissues underwent dehydration and embedding in paraffin to maintain structural integrity. Sections were cut into thin slices of approximately 5 μm and stained using Sirius red staining to evaluate general histological features, and Masson’s trichrome staining to highlight collagen distribution, which is critical in assessing myocardial fibrosis [12]. The presence of collagen within myocardial tissues, indicating fibrotic remodeling, was quantified by measuring the area of collagen deposition in relation to the total myocardial area, following established procedures in myocardial tissue analysis [8]. Images of the stained sections were obtained using a Leica DM4000B light microscope (Leica Microsystems, Wetzlar, Germany), allowing for a clear visualization of histopathological features. These images provided a basis for quantitative analysis, wherein the myocardial damage was assessed by calculating the ratio of the area infiltrated by inflammatory cells to the total myocardial area. Similarly, collagen deposition, a marker of fibrosis, was quantified to evaluate the progression of fibrotic remodeling. This assessment was executed with Image Pro Plus software (version 6.0; Media Cybernetics, Bethesda, MD, USA), which facilitated precise measurement of both inflammatory and fibrotic changes within the cardiac tissue. The quantitative evaluation of myocardial tissue using Sirius red staining and Masson’s trichrome staining offers insights into the extent of histological alterations and provides a framework to interpret anti-inflammatory and anti-fibrotic interventions in cardiovascular pathology.

### 2.5. Western Blot Analysis

Heart tissue was collected from animal models, immediately rinsed with ice-cold PBS to maintain integrity, and lysed using radioimmunoprecipitation assay (RIPA) buffer. The buffer was enriched with protease inhibitors, including phenylmethylsulfonyl fluoride and phosphatase inhibitors, to protect against protein degradation, as seen in methodologies for examining protein activity under various inflammatory conditions [8]. Protein concentrations in the lysates were quantified with a Bicinchoninic Acid Protein Assay Kit, ensuring accurate measurements for consistent loading [11]. Following quantification, equal protein amounts were loaded onto a 10% SDS-PAGE gel for separation by electrophoresis, a standard technique used to resolve proteins by molecular weight. After electrophoresis, proteins were transferred onto polyvinylidene fluoride (PVDF) membranes, optimizing binding efficiency for subsequent detection [10]. To minimize nonspecific antibody interactions, membranes were blocked in a 5% non-fat milk solution for 90 min, which serves as a general protein blocker. Primary antibodies targeting specific proteins associated with cardiac function and inflammation, such as Gal-3 (1:2000), CD68, CTGF, DDR2, alpha-SAM, CD44, and collagen I (all at 1:1000), were diluted in Tris-buffered saline with Tween-20 (TBST), and applied overnight at 4 °C. Each primary antibody was selected based on its relevance to the pathways of interest in inflammation and fibrosis, as shown in related studies of cardiovascular markers [13]. GAPDH (1:10,000) was used as a housekeeping protein for normalization, providing a control for equal protein loading across samples. Following overnight incubation, membranes were washed with TBST to eliminate excess unbound primary antibodies, then incubated with horseradish peroxidase (HRP)-conjugated secondary antibodies at a dilution of 1:20,000 for one hour. The HRP enzyme on the secondary antibody allows for chemiluminescent detection of target proteins when combined with an enhanced chemiluminescence (ECL) substrate [9]. Protein bands were visualized using the Fusion FX5 Spectra imaging system (Vilber Lourmat, Collégien, France), a highly sensitive method for capturing low-abundance proteins.

### 2.6. Enzyme-Linked Immunosorbent Assay

On day 22 of the experiment, blood samples were collected and centrifuged to separate supernatants. The serum was then added to the wells and incubated with enzyme-conjugated anti-Gal-3 antibodies (R&D, Cat number MAB1197). Unbound conjugate was removed by washing, and an enzyme substrate was added to each well. The color generated was proportional to the amount of Gal-3 in the serum. Absorbance was measured immediately using an ELISA reader (TECAN, Infinite 200 Pro) at 450 nm.

### 2.7. Statistical Analysis

Data analysis was conducted using SPSS 22.0 software (SPSS Inc., Chicago, IL, USA) and GraphPad Prism 8 software (GraphPad, San Diego, CA, USA). The results are presented as mean ± standard deviation (SD). Normality of the data was verified, after which one-way analysis of variance (ANOVA) was used to determine statistical significance among groups. For pairwise group comparisons, post hoc multiple comparison testing was employed, with a *p*-value < 0.05 considered statistically significant.

## 3. Results

This section provides a detailed analysis of the experimental outcomes from fucoidan administration in a mouse model of TAC-induced cardiac hypertrophy. The results include morphological, functional, and biochemical assessments to elucidate fucoidan’s impact on cardiac structure and fibrosis-related pathways.

### 3.1. Fucoidan Reduces Heart and Left Ventricular Weights in TAC-Induced Cardiac Hypertrophy

#### Cardiac Morphology and Weight Analysis

**Figure 2:**
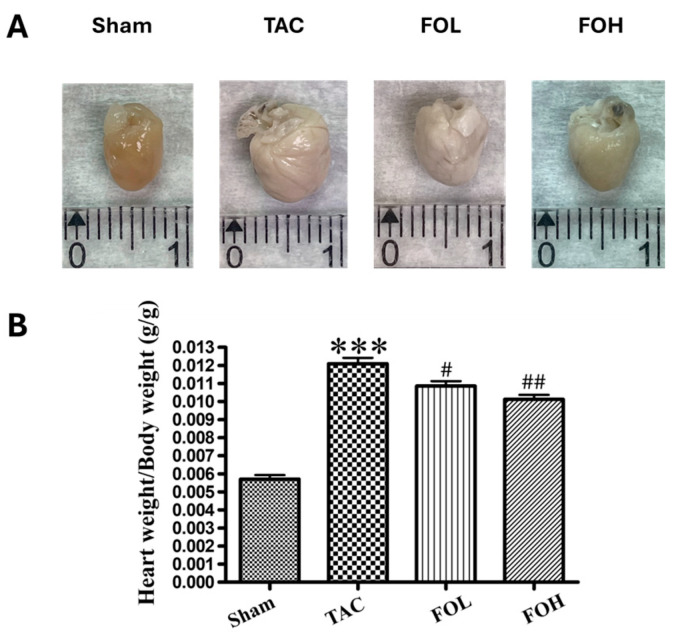
Effects of Fucoidan on whole heart and left ventricular tissue weights, as well as on body weight under varying experimental conditions. (**A**) Heart images from each experimental group visually depict the impact of Fucoidan treatment. (**B**) The heart weight-to-body weight ratio demonstrates significant variations across groups, with TAC surgery inducing notable increases in both metrics. Fucoidan treatment, however, reduced these ratios compared to the TAC-induced cardiac hypertrophy group. Experimental groups included the sham-operated control (sham), TAC-induced cardiac hypertrophy group (TAC), TAC group treated with 1.5 mg/kg/day Fucoidan (FOL), and TAC group treated with 7.5 mg/kg/day Fucoidan (FOH). Data are presented as means ± standard deviation (SD) with *n* = 5. *** *p* < 0.001 vs. sham; and ^#^ *p* < 0.05 and ^##^ *p* < 0.01 vs. TAC.

The administration of fucoidan showed a notable effect on heart and left ventricular weights in TAC-induced hypertrophy models. Figure 2A shows representative images of whole-heart specimens from different groups, illustrating fucoidan’s influence on cardiac morphology. TAC surgery significantly increased heart and left ventricular weights (Figure 2A,B). However, in the fucoidan-treated TAC group, these weights were reduced compared to the untreated TAC group, indicating an attenuation of hypertrophy. These findings suggest fucoidan’s potential as a therapeutic agent for cardiac hypertrophy, likely due to its roles in modulating oxidative stress and inflammation, thereby counteracting hypertrophic remodeling.

### 3.2. Fucoidan Enhances Cardiac Function in TAC-Induced Cardiac Hypertrophy

#### 3.2.1. Echocardiographic Analysis of Cardiac Function

**Figure 3:**
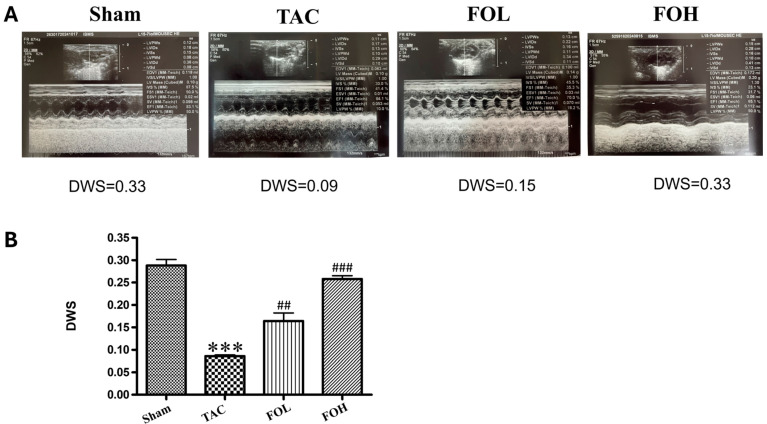
Effects of Fucoidan on cardiac function in a mouse model of transverse aortic constriction (TAC)-induced cardiac hypertrophy. (**A**) Representative echocardiographic images from each experimental group illustrate changes in cardiac function. (**B**) Analysis of diastolic wall strain (DWS) across groups reveals significant differences. The TAC-induced cardiac hypertrophy group exhibited a reduced ejection fraction compared to the sham-operated control, while Fucoidan treatment partially restored the ejection fraction in both treatment groups. DWS, calculated from posterior wall thickness (PWT) measurements as DWS = [PWT(systole) − PWT(diastole)]/PWT(systole), serves as a marker for left ventricular (LV) diastolic stiffness. The experimental groups included sham-operated controls (sham), the TAC-induced hypertrophy group (TAC), and TAC groups treated with 1.5 mg/kg/day (FOL) or 7.5 mg/kg/day Fucoidan (FOH). Data are expressed as means ± standard deviation (SD), with *n* = 5. Statistical significance is indicated by and *** *p* < 0.001 vs. sham, and ^##^ *p* < 0.01, and ^###^ *p* < 0.001 vs. TAC.

Echocardiographic evaluations revealed differences in structural parameters between the experimental groups (Figure 3A), with fucoidan-treated groups showing improvement in several indicators of cardiac performance. Key parameters, such as diastolic wall strain (DWS), improved significantly with fucoidan treatment, along with partial restoration of ejection fraction in the treated TAC group compared to untreated TAC animals (Figure 3B). Consistent with Zhang et al. (2024) [11], who observed that fucoidan reduced myocardial damage in inflammation-induced models, these findings suggest that fucoidan’s anti-inflammatory and antioxidative properties play a role in cardiac protection during hypertrophic stress.

#### 3.2.2. Ventricular Volume and Dimension Parameters

Analysis of left ventricular end-diastolic volume (LVEDV) and end-systolic volume (LVESV) revealed significant increases in the TAC group compared to the Sham group, reflecting ventricular dilation and impaired cardiac function. Fucoidan treatment (both FOL and FOH groups) significantly reduced these volumes, suggesting attenuation of maladaptive ventricular remodeling (Table 1). Importantly, the ejection fraction (EF), a critical measure of cardiac function, was significantly reduced in the TAC group compared to the Sham group, indicating systolic dysfunction. Fucoidan treatment partially restored EF, with the FOH group demonstrating greater improvement than the FOL group. This improvement in EF supports the hypothesis that fucoidan treatment enhances cardiac function in addition to reducing structural remodeling. Furthermore, fucoidan treatment showed trends toward normalization in left ventricular internal dimensions at end-systole (LVIDs) and end-diastole (LVIDd) periods, as well as left ventricular posterior wall thickness (LVPWd). Together, these results highlight the therapeutic potential of fucoidan in mitigating structural and functional impairments associated with cardiac hypertrophy. These observations align with studies by Wang et al. (2024) [9], who noted fucoidan’s role in maintaining cardiovascular integrity through enhanced endothelial compatibility and reduced oxidative stress.

#### 3.2.3. Interventricular Measurements

Measurements of interventricular septal thickness at end-diastole (IVSd) and end-systole (IVSs) showed minimal variation across groups (Table 1). This suggests that fucoidan’s effects on cardiac remodeling and function are region-specific, primarily influencing ventricular dilation and systolic performance. The spatial specificity of fucoidan’s effect is consistent with findings by Park et al. (2024) [13], which highlighted localized actions of fucoidan in inflammatory and stress-related cardiac conditions.

### 3.3. Fucoidan Attenuates Histopathological Damage in TAC-Induced Hypertrophy

#### 3.3.1. Histological Analysis of Myocardial and Collagen Accumulation

**Figure 4:**
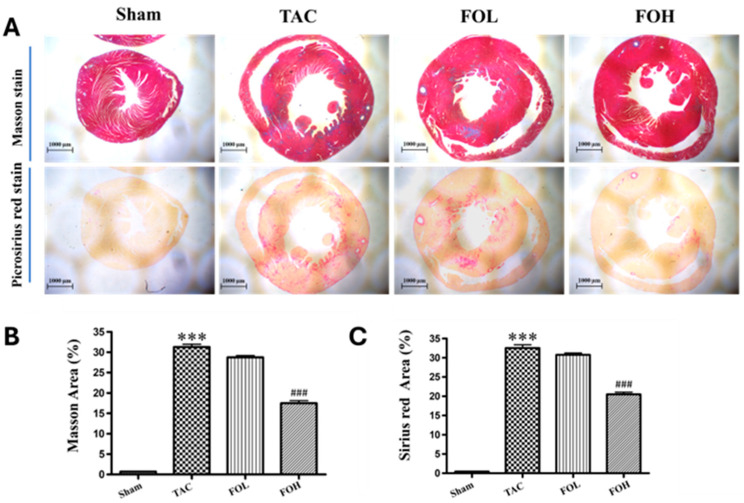
Effects of Fucoidan on histopathological changes in mouse heart tissue. (**A**) Representative images of Masson staining (top panels) and Sirius red staining (bottom panels) of left ventricular tissue from each group are shown at 200× magnification, with scale bars indicating 1000 μm. (**B**,**C**) Quantitative analyses display the area percentage of myocardial damage and collagen deposition (*n* = 5), with data presented as mean ± standard deviation (SD). Statistical significance is noted as *** *p* < 0.001, compared to the control sham group, and ^###^ *p* < 0.001, compared to the TAC group.

Histological staining (Masson’s trichrome and Sirius red) revealed extensive myocardial inflammation, necrosis, and structural disarray in the TAC group, as well as significant collagen deposition, indicating fibrosis (Figure 4). Fucoidan treatment reduced these pathological changes, including collagen accumulation in the left ventricle, as indicated by a decreased area percentage of fibrosis, suggesting a protective role against fibrosis. These results align with Ersoydan and Rustemeyer (2024), who reported the anti-inflammatory effects of fucoidan [8], and Zhang et al. (2024), who noted its antioxidative properties in myocardial damage models [11].

#### 3.3.2. Implications for Cardiac Fibrosis

The reductions in collagen deposition and myocardial disarray underscore fucoidan’s ability to counter fibrosis and support structural integrity in hypertrophic cardiac settings. This effect may result from fucoidan’s modulation of both inflammatory and antioxidative pathways, as previously noted in cardiovascular studies by Wang et al. (2024) [9].

### 3.4. Fucoidan Reduces Galectin-3 Levels in Pressure Overload-Induced Cardiac Remodeling

#### Galectin-3 (Gal-3) Analysis by ELISA

**Figure 5:**
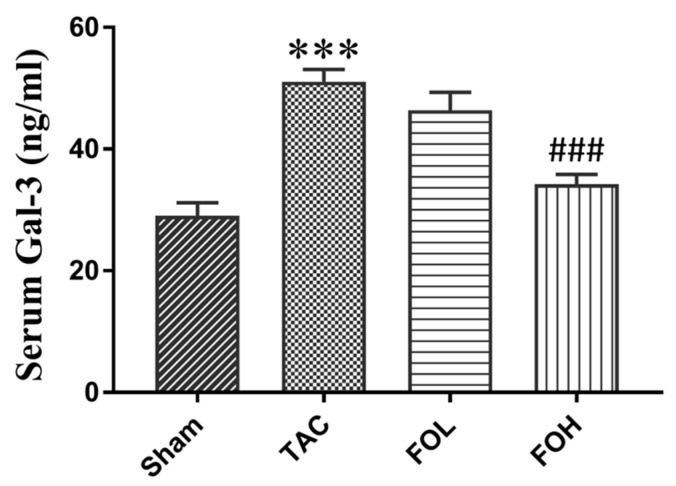
Effect of fucoidan on serum Galectin-3 (Gal-3) levels in mice following transverse aortic constriction (TAC). Serum Gal-3 levels were measured by enzyme-linked immunosorbent assay (ELISA) at day 22 post-TAC. The TAC group exhibited a significant increase in Gal-3 levels compared to the sham group, indicating fibrosis due to pressure overload. Treatment groups receiving fucoidan (FOL and FOH) showed a marked reduction in Gal-3 levels compared to the TAC group, suggesting fucoidan’s potential to mitigate fibrosis-related signaling. Data are expressed as mean ± standard deviation (SD) (*n* = 5). Statistical significance is indicated as *** *p* < 0.001 vs. sham, and ^###^ *p* < 0.001 vs. TAC.

ELISA measurements of Gal-3 levels in the serum indicated a significant increase in TAC-induced cardiac hypertrophy, associated with fibrosis onset (Figure 5). Fucoidan treatment led to reduced Gal-3 accumulation in the intervention groups, suggesting that fucoidan may counter fibrotic signaling through Gal-3 downregulation, potentially alleviating adverse remodeling under pressure overload conditions.

### 3.5. Fucoidan Modulates Inflammatory and Fibrotic Signaling Pathways

#### 3.5.1. Western Blot Analysis of Fibrotic Markers

**Figure 6:**
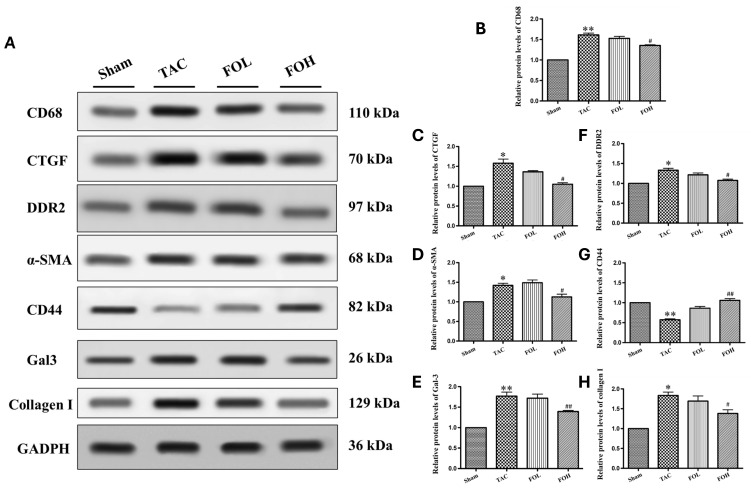
Fucoidan modulates key inflammatory and fibrotic signaling pathways, highlighting its potential as a therapeutic candidate for cardiovascular and fibrotic diseases. This study evaluates the effects of fucoidan on pro-inflammatory and fibrotic signaling pathways using Western blot analysis to detect specific markers associated with inflammation and fibrosis. The markers analyzed include Galectin-3 (Gal-3), CD68, connective tissue growth factor (CTGF), discoidin domain receptor 2 (DDR2), alpha-smooth muscle actin (α-SMA), CD44, and collagen I, providing insights into fucoidan’s regulatory effects on these pathways. (**A**) Protein levels of CD68, CTGF, DDR2, α-SMA, CD44, and collagen I in each group on day 22 are shown. (**B**–**H**) Quantification of protein bands for CD68, CTGF, DDR2, α-SMA, CD44, and collagen I, normalized to GAPDH as the internal control (*n* = 3). Data are presented as mean ± standard deviation (SD). Statistical significance is indicated as follows: * *p* < 0.05 vs. sham group; ** *p* < 0.01 vs. sham group; ^#^ *p* < 0.05 vs. TAC group; ^##^ *p* < 0.01 vs. TAC group.

Western blot analysis of markers involved in fibrosis and inflammation, including Gal-3, CD68, CTGF, DDR2, α-SMA, and collagen I, showed significant increases post-TAC surgery (Figure 6). Fucoidan treatment, however, resulted in reduced expression of these proteins, indicating attenuation of pro-inflammatory and pro-fibrotic responses. Notably, fucoidan restored CD44 expression in TAC-induced models, suggesting a specific mechanism by which it may counter fibrosis through receptor modulation, as CD44 is involved in cellular adhesion and migration essential to tissue repair [10].

#### 3.5.2. Overall Pathway Modulation by Fucoidan

Fucoidan’s multifaceted effects on inflammation and fibrosis are reflected in its ability to downregulate multiple pathways linked to cellular stress and remodeling. By reducing markers such as Gal-3, CD68, CTGF, DDR2, α-SMA, and collagen I (Figure 6B–F,H), while modulating CD44 (Figure 6G), fucoidan appears to counteract the progression of hypertrophy through anti-inflammatory and antioxidative mechanisms. These results are consistent with previous studies that highlight fucoidan’s capacity to mitigate inflammatory and fibrotic responses, supporting its potential as a therapeutic agent for managing hypertrophic cardiac conditions through comprehensive pathway modulation.

## 4. Discussion

**Figure 7:**
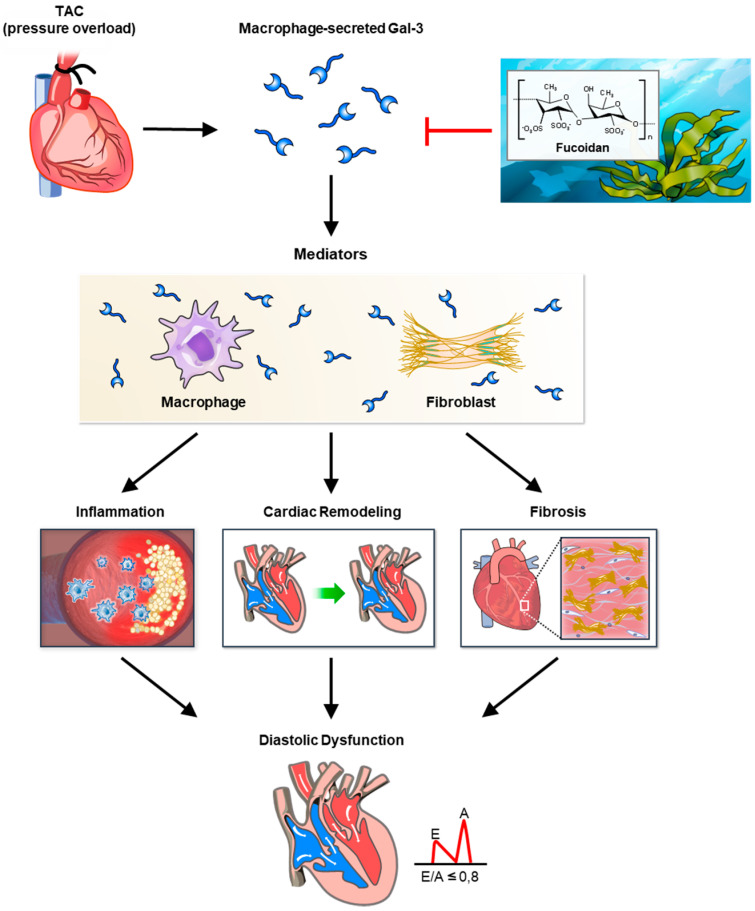
Summary of fucoidan’s effects on cardiac remodeling and inflammation in pressure overload-induced cardiac hypertrophy. This figure illustrates the protective effects of fucoidan against cardiac remodeling, fibrosis, and inflammation in a mouse model of pressure overload-induced cardiac hypertrophy generated by transverse aortic constriction (TAC). TAC surgery was used to induce pressure overload, resulting in pathological cardiac hypertrophy. Following aortic constriction, there was a significant increase in cardiac Galectin-3 (Gal-3) expression, a protein closely associated with fibrosis and inflammation. Elevated Gal-3 levels correlated with increased collagen deposition and upregulation of fibrotic mediators, indicative of cardiac fibrosis. In addition, TAC-induced pressure overload led to increased levels of inflammatory markers and a marked infiltration of inflammatory cells into cardiac tissue, reflecting an inflammatory response associated with cardiac hypertrophy. Fucoidan treatment effectively mitigated the adverse effects of pressure overload. Fucoidan administration prevented the upregulation of Gal-3, resulting in decreased collagen deposition and downregulation of fibrotic mediators, indicating attenuation of cardiac fibrosis. Additionally, fucoidan significantly reduced inflammatory cell infiltration and lowered inflammatory cytokine levels in TAC mice, highlighting its anti-inflammatory effects. These findings suggest that fucoidan can delay or prevent the progression of cardiac remodeling in pressure overload by inhibiting Gal-3, reducing fibrosis, and decreasing inflammation. Fucoidan shows promise as a therapeutic agent for mitigating Gal-3 overexpression and associated cardiac complications. The arrow indicates the direction of the observed effect, while the red hammer symbolizes the inhibitory effect.

In this study, fucoidan was shown to significantly reduce Gal-3 expression, fibrosis, and inflammation in a mouse model of pressure overload, illustrating its potential therapeutic effects against cardiac remodeling (Figure 7). The findings indicate that fucoidan’s suppression of Gal-3, a protein strongly associated with fibrosis and inflammation, likely mitigates fibrotic processes in the myocardium, reducing the extent of structural remodeling under pressure overload conditions. Notably, previous research supports these anti-inflammatory and antifibrotic effects, as demonstrated by fucoidan’s modulation of immune responses and its influence on inflammation-related signaling pathways, including CD44 and Nrf2/GPX4, which are crucial for cellular protection under oxidative stress conditions. Fucoidan’s effect on inflammation was also apparent through its reduction in markers associated with immune cell infiltration and fibrotic signaling. Studies on high-molecular-weight fucoidan, such as those by Park et al. (2024) [13], highlight its immune-enhancing properties, which might contribute to the attenuation of cardiac inflammation observed in this model. Additionally, work by Zhang et al. (2024) underscores fucoidan’s capacity to downregulate lipopolysaccharide-induced inflammatory markers [11], suggesting a broad anti-inflammatory role in cardiovascular settings. These results position fucoidan as a promising agent for controlling cardiac hypertrophy and fibrosis by targeting multifaceted mechanisms involved in inflammatory and fibrotic signaling pathways.

Fucoidan’s cardioprotective effects in pressure-overload conditions appear to involve key antioxidative and anti-inflammatory pathways that modulate Gal-3 and fibrosis-related markers. One critical mechanism includes the activation of the Nrf2 pathway, which enhances cellular defenses against oxidative stress. By upregulating Nrf2 signaling, fucoidan helps increase the expression of antioxidant enzymes, thereby reducing oxidative damage—a factor that can drive fibrosis and inflammation in cardiac tissue. Studies demonstrate that Nrf2 activation also plays a role in inhibiting ferroptosis, a form of cell death linked to cardiac remodeling, which may further contribute to Gal-3 modulation in fucoidan-treated hearts [9,14]. Furthermore, fucoidan’s anti-inflammatory properties may counteract fibrosis progression by modulating the CD44 pathway, a receptor associated with inflammation-induced tissue changes. Research by Chen et al. (2024) suggests that pressure-induced fibrosis and inflammation involve CD44 signaling, which is downregulated by fucoidan [10]. This modulation likely inhibits pathways leading to Gal-3 upregulation, reducing the pro-fibrotic responses in cardiac tissue. Studies on fucoidan’s action in immune cells have also highlighted its ability to decrease inflammatory mediators [11,13], suggesting a broader role in attenuating inflammatory pathways central to cardiac fibrosis. The reduction in Gal-3 secretion and fibrosis markers indicates that fucoidan may limit oxidative stress and inflammatory signals that exacerbate remodeling. Its influence on these molecular targets presents a multifaceted approach to mitigating cardiac remodeling, supporting the compound’s potential as a therapeutic intervention against fibrosis in pressure-overload contexts.

This study aligns with prior research on fucoidan’s cardioprotective effects but extends the understanding of its potential by demonstrating a more direct impact on Gal-3 expression and fibrosis in pressure overload-induced cardiac remodeling. Previous studies have shown that fucoidan from various brown algae, such as those explored by Ersoydan and Rustemeyer (2024) [8], can inhibit inflammation and reduce oxidative stress in cardiovascular models, suggesting a broad anti-inflammatory and antioxidant role. However, while earlier research focused on general anti-inflammatory actions, the current findings specifically indicate that fucoidan suppresses Gal-3 expression, a key fibrosis-associated protein. This adds to the literature by highlighting Gal-3 as a targeted mechanism in fucoidan’s antifibrotic actions. In addition, studies by Zhang et al. (2024) demonstrated that fucoidan from Costaria costata mitigates inflammation in lipopolysaccharide-induced mouse models, which supports the notion that fucoidan’s immunomodulatory effects contribute to cardiac resilience [11]. Unlike Zhang’s work, which primarily addresses systemic inflammation, this study focuses on cardiac-specific fibrotic and inflammatory pathways, suggesting that fucoidan’s effects may be particularly beneficial for preventing fibrosis in hypertrophic cardiac conditions. Furthermore, other studies on fucoidan-loaded hydrogel coatings, such as those by Wang et al. (2024), highlight the compound’s capacity to promote endothelial growth and improve cardiovascular outcomes through antioxidant mechanisms, particularly through the Nrf2/GPX4 pathway [9]. The present study not only confirms these antioxidative pathways but also specifies that fucoidan’s Nrf2-related antioxidative action likely inhibits Gal-3, thereby reducing fibrotic remodeling in pressure-overloaded myocardium. This insight advances our understanding of how fucoidan can specifically prevent cardiac hypertrophy by integrating antioxidative and antifibrotic effects focused on Gal-3 modulation. Collectively, this study contributes to the existing literature by reinforcing fucoidan’s utility in fibrosis inhibition while providing novel mechanistic insights into its ability to directly target Gal-3. This potentially positions fucoidan as an essential therapeutic option for cardiac hypertrophy, particularly in conditions marked by inflammation and oxidative stress.

Fucoidan shows promising therapeutic potential for conditions associated with cardiac hypertrophy and fibrosis. Its ability to modulate multiple cellular pathways, such as reducing oxidative stress via the Nrf2 pathway and dampening inflammation through CD44-related mechanisms, indicates a comprehensive impact on processes underlying cardiac remodeling. By inhibiting Ga-3 and thereby reducing fibrotic activity, fucoidan could directly address fibrosis, a key factor in cardiac dysfunction in hypertrophic hearts [10,14]. Therapeutically, fucoidan’s multi-targeted effects make it especially relevant for cardiovascular diseases where inflammation and oxidative stress are prominent. Research by Wang et al. (2024) illustrates that fucoidan’s antioxidative effects extend beyond simple antioxidant activity [9], engaging cellular defenses to improve mitochondrial resilience and reduce apoptosis, which is critical for preserving cardiac tissue under stress. Moreover, studies on high-molecular-weight fucoidan suggest immune-enhancing effects, indicating that it may strengthen the body’s overall defense mechanisms, which are often compromised by chronic inflammatory states associated with heart disease [13]. Fucoidan’s broad therapeutic potential makes it a promising candidate for managing cardiac hypertrophy and fibrosis, especially in clinical settings where oxidative stress and inflammation exacerbate disease progression. Its ability to modulate key pathways such as Keap1/Nrf2 and CD44 contributes not only to reduction in fibrosis markers like Gal-3 but also to systemic antioxidative and anti-inflammatory defenses, which are critical for maintaining cardiac health [10,14]. In clinical practice, these properties could translate into meaningful therapeutic outcomes. For instance, by enhancing the antioxidative response via the Nrf2 pathway, fucoidan may help protect cardiomyocytes and other cardiac cells from oxidative damage, which is a key driver of maladaptive remodeling and heart failure. Studies have demonstrated that this pathway plays a role in mitigating ferroptosis—a type of programmed cell death linked to cardiac injury—highlighting its potential to preserve cardiac function [9,11]. Also, fucoidan’s influence on CD44 signaling, which mediates inflammation-induced fibrosis, suggests its utility in reducing structural and functional deterioration in conditions of chronic pressure overload [10]. By targeting CD44 and Gal-3 simultaneously, fucoidan may disrupt the feedback loops between inflammation and fibrosis, preventing progressive damage to cardiac tissue. Fucoidan’s benefits extend beyond direct cardiac effects. Its broad-spectrum immune-enhancing properties, as shown in both preclinical and clinical studies, indicate its potential to improve systemic health, particularly in patients with comorbid conditions such as diabetes or metabolic syndrome, which often exacerbate cardiac pathology [13,15]. For example, its ability to modulate gut microbiota and enhance systemic antioxidative capacity could complement its cardioprotective effects, creating a multifaceted therapeutic approach [16]. From a translational perspective, fucoidan could serve as an adjunctive therapy to standard treatments for heart failure and other cardiovascular diseases, particularly those characterized by inflammatory and oxidative stress components. Its incorporation into advanced delivery systems, such as hydrogel coatings for cardiovascular stents, has already shown promise in improving biocompatibility and promoting vascular healing, underscoring its versatility in various clinical applications [17]. Further clinical studies are needed to establish optimal dosing regimens, delivery methods, and long-term safety profiles for fucoidan. Nonetheless, its ability to address multiple pathways involved in cardiac remodeling positions it as a valuable addition to the arsenal of therapies for hypertrophic and fibrotic cardiovascular diseases.

This study has several limitations that should be acknowledged. Firstly, the absence of a sham group with fucoidan supplementation limits our ability to evaluate its basal effects on cardiac health. While this was not included in the current experimental design, future studies should incorporate such a group to provide insights into the baseline impact of fucoidan, independent of stress conditions. Evidence from prior research suggests that fucoidan exerts anti-inflammatory and antioxidative effects, even under non-stressed conditions, potentially enhancing cardiac and systemic health by modulating gut microbiota and reducing systemic inflammation [11,13]. Secondly, while this study employed the TAC model to induce pressure overload, we did not measure the pressure gradient across the aortic constriction at key time points. This represents a limitation in ensuring consistency in the severity of pressure overload among groups. Future experiments should include pressure gradient measurements both one week post-surgery and before the final sacrifice, as this would verify the uniformity of the model and improve its robustness. Prior studies have emphasized the importance of such assessments in correlating hemodynamic parameters with structural and molecular changes in cardiac remodeling [18]. Thirdly, this study did not employ wheat germ agglutinin (WGA) staining to assess cardiomyocyte hypertrophy or terminal deoxynucleotidyl transferase dUTP nick end labeling (TUNEL) staining to quantify apoptosis. Both methods could have provided additional cellular context to the observed effects of fucoidan. Future studies should include these techniques to validate and extend our findings, particularly in light of evidence showing fucoidan’s protective effects against hypertrophy and apoptosis via pathways like Nrf2/GPX4 and Notch signaling [9,16]. Fourthly, the causal relationship between fucoidan’s effects and Gal-3 suppression was not established in this study. While our results demonstrate a correlation, further mechanistic studies using Gal-3 supplementation or silencing in the presence and absence of fucoidan are necessary to clarify whether Gal-3 suppression is a direct or indirect effect mediated by other signaling proteins. Such experiments in isolated cardiomyocytes or fibroblasts would provide definitive insights into fucoidan’s molecular mechanisms [10]. Fifthly, the use of an animal model, while valuable for preclinical investigation, poses challenges in translating findings to human applications. Variability in metabolism, physiological response, and immune modulation between species could influence how fucoidan behaves in human systems compared to mice [8]. Additionally, dosage levels used in animal studies may not correspond linearly to safe and effective dosages in humans, necessitating careful recalibration through clinical trials [9,11]. Another limitation relates to the bioavailability of fucoidan when administered in different forms. While fucoidan-loaded hydrogel coatings and oral supplementation have shown promise in experimental settings, differences in absorption, distribution, and degradation in human systems may affect efficacy and dosing requirements [10]. Lastly, while this study focused on specific markers such as Gal-3, a more comprehensive approach examining additional inflammatory and oxidative markers would further clarify fucoidan’s therapeutic potential and inform its broader applicability in human cardiology.

Future research should prioritize elucidating the molecular mechanisms by which fucoidan influences cardiac remodeling, with particular focus on its effects on cardiomyocytes and fibroblasts. While in vitro experiments on isolated cells were beyond the scope of the current study, incorporating such models in future studies could enhance understanding of fucoidan’s cellular targets. Specifically, cultured neonatal rat cardiomyocytes or fibroblasts, or cells isolated from adult hearts, could be valuable tools for this purpose. One promising avenue is the interaction between Gal-3 and interleukin-1 receptor like-1 (IL-1RL1), also known as suppression of tumorigenicity 2 (ST2), a receptor for IL-33. The soluble form, sST2, is a critical mediator of cardiac fibrosis and inflammation, serving as a biomarker for heart failure severity [18]. Elevated sST2 inhibits the protective IL-33/transmembrane form ST2 (ST2L) signaling axis, exacerbating maladaptive remodeling. Fucoidan’s effects on Gal-3 expression suggest it may attenuate this pathological cascade. Future studies should explore whether fucoidan modulates sST2 levels or restores IL-33/ST2L signaling, potentially amplifying its cardioprotective effects. Mechanistically, fucoidan’s antioxidative properties, mediated through the Nrf2/GPX4 pathway, and its anti-inflammatory effects, potentially through CD44 signaling, could indirectly downregulate sST2 by mitigating upstream oxidative and inflammatory triggers [9,10]. These pathways are central to cardiac fibrosis and remodeling, and their modulation by fucoidan aligns with findings from studies highlighting its ability to suppress lipid accumulation, enhance mitochondrial function, and regulate gut microbiota-related inflammation [11,14]. The antifibrotic effects of fucoidan may extend to its potential to influence fibroblast proliferation and extracellular matrix production. Research indicates that fucoidan can suppress fibrosis-related pathways via Notch signaling and reduce oxidative damage in various models [15,16]. Furthermore, its bioactivity in altering macrophage polarization and reducing inflammatory cytokines highlights its broader immunomodulatory role [11,13]. For clinical translation, studies should assess changes in sST2 and Gal-3 levels following fucoidan treatment in animal models of heart failure. Preclinical experiments could include hydrogel-based controlled-release systems to improve the bioavailability and sustained therapeutic effects of fucoidan [17]. Such systems could be particularly effective in modulating both Gal-3 and ST2 pathways, as demonstrated by advances in cardiovascular biomaterials. Finally, clinical trials are necessary to evaluate fucoidan’s long-term effects on cardiac remodeling and function, particularly in patients with elevated sST2 and Gal-3 levels. Investigating the synergy of targeting both pathways could reveal new dual-targeted therapeutic strategies. By integrating these molecular and cellular insights, future research will better define fucoidan’s potential as a multifaceted treatment for heart failure, addressing the interplay between fibrosis, oxidative stress, and inflammation.

## 5. Conclusions

This study demonstrates that fucoidan effectively mitigates cardiac remodeling in a TAC-induced mouse model of pressure overload. Fucoidan treatment significantly reduced cardiac hypertrophy and fibrosis, as evidenced by decreased heart weight, improved myocardial structure, and reduced markers of oxidative stress, inflammation, and fibrosis, including Gal-3, α-SMA, and collagen I. Furthermore, fucoidan’s modulation of key signaling pathways, such as Keap1/Nrf2 and CD44, highlights its antioxidative and anti-inflammatory properties, which are critical in protecting cardiac tissue under stress [9,10]. Histological analysis confirmed reduced collagen deposition and improved myocardial organization, suggesting that fucoidan preserves cardiac structure and function. Improvements in cardiac parameters, such as ejection fraction and ventricular volumes, further underscore its potential to delay the progression of heart failure. These findings provide strong evidence that fucoidan’s multifaceted effects on oxidative stress, inflammation, and fibrosis position it as a promising therapeutic candidate for conditions characterized by pathological cardiac remodeling. Future research should focus on elucidating the precise molecular mechanisms underlying fucoidan’s cardioprotective effects, particularly its interactions with fibrosis and inflammation pathways. Moreover, clinical studies are needed to evaluate its efficacy and safety in human populations, particularly in hypertrophic and fibrotic cardiac diseases where oxidative stress and inflammation play a central role [8,11].

## Figures and Tables

**Table 1:** Summarizes the impact of fucoidan treatment on echocardiography parameters in mice with transverse aortic constriction (TAC)-induced cardiac hypertrophy.

Empty Cell	Sham	TAC	FOL	FOH
LVEDV (mL)	0.129 ± 0.038	0.172 ± 0.063 **	0.210 ± 0.072 ^##^	0.220 ± 0.038 ^##^
LVESV (mL)	0.028 ± 0.017	0.054 ± 0.027 **	0.076 ± 0.007 ^##^	0.076 ± 0.015 ^##^
SV (mL)	0.101± 0.024	0.118 ± 0.037 **	0.126 ± 0.009 ^#^	0.144 ± 0.023 ^##^
LVd Mass	0.102 ± 0.012	0.218 ± 0.069 **	0.190 ± 0.040 ^#^	0.204 ± 0.021 ^#^
LVIDs	0.210 ± 0.046	0.266 ± 0.054 **	0.296± 0.030 ^#^	0.308 ± 0.024 ^##^
LVIDd	0.368 ± 0.035	0.402 ± 0.061 **	0.422 ± 0.075 ^#^	0.446 ± 0.027 ^##^
LVPWd	0.078 ± 0.004	0.132 ± 0.035 *	0.100 ± 0.005 ^#^	0.100 ± 0.000 ^#^
IVSs	0.124 ± 0.019	0.142 ± 0.016	0.136 ± 0.010	0.134 ± 0.021
IVSd	0.080 ± 0.000	0.128 ± 0.017	0.108 ± 0.010	0.110 ± 0.011
EF	78.29 ± 4.35	68.60 ± 3.85 **	71.50 ± 3.20 ^#^	74.50 ± 2.95 ^##^

Table 1 provides a summary of the effects of Fucoidan treatment on echocardiographic parameters in mice with cardiac hypertrophy induced by transverse aortic constriction (TAC). The parameters evaluated include the following: LVEDV: left ventricular end-diastolic volume; LVESV: left ventricular end-systolic volume; SV: stroke volume; LVd Mass: left ventricular diastolic mass; LVIDs: left ventricular internal dimension in systole; LVIDd: left ventricular internal dimension in diastole; LVPWd: left ventricular posterior wall thickness at end diastole; IVSs: interventricular septum thickness in systole; IVSd: interventricular septum thickness at end diastole; EF: ejection fraction. Data are presented as means ± standard deviation (SD) with a sample size of *n* = 6. Statistical significance is indicated by * *p* < 0.05 and ** *p* < 0.01 compared to the Sham group, and by ^#^ *p* < 0.05, ^##^ *p* < 0.01 compared to the TAC group.

## Data Availability

The data supporting the findings of this study can be accessed upon reasonable request from the corresponding author. Requests will be evaluated in accordance with MDPI’s Research Data Policies.
